# A Case of Fulminant Type 1 Diabetes With Residual Insulin Secretion

**DOI:** 10.7759/cureus.82751

**Published:** 2025-04-21

**Authors:** Tomoko Kato, Norio Harada, Yuki Nishikawa, Kana Yamamoto, Takaaki Murakami, Yohei Ueda, Motozumi Okamoto, Daisuke Yabe

**Affiliations:** 1 Diabetes, Endocrinology, and Nutrition, Graduate School of Medicine, Kyoto University, Kyoto, JPN; 2 Endocrinology and Metabolism, University of Fukui School of Medical Sciences, Fukui, JPN; 3 Internal Medicine, Otsu-Shiga Red Cross Hospital, Otsu, JPN

**Keywords:** fulminant type 1 diabetes, insulin secretory ability, ketosis, residual insulin secretion, type 1 diabetes

## Abstract

A 67-year-old woman presented with fever and a cold a week later, with symptoms of hyperglycemia. After 10 days, her non-fasting blood glucose level was 586 mg/dL, and glycosylated hemoglobin (HbA1c) was 7.2%. She then developed diabetic ketosis and was treated with insulin. Her fasting C-peptide immunoreactivity (CPR) became 0.4 ng/mL. However, her insulin requirement was subsequently reduced. After two months, her CPR was 1.07 ng/mL at zero minutes and 2.04 ng/mL at six minutes in a glucagon stimulation test. Daily urinary CPR was 62 mg. Even though fulminant type 1 diabetes was indicated from the clinical course, insulin secretory ability improved.

## Introduction

Fulminant type 1 diabetes is a subtype of type 1 diabetes characterized by the rapid progression of pancreatic β-cell destruction and insulin secretory disorder [1]. The people with fulminant type 1 diabetes develop a markedly rapid onset of hyperglycemia with ketoacidosis. The following findings are also clinical characteristics observed in this subtype: the negative status of islet‐related autoantibodies, elevation of serum pancreatic enzyme levels, presence of prodrome such as flu-like symptoms or gastrointestinal symptoms, and association with human leukocyte antigen (HLA) DRB1*04:05-DQB1*04:01 [1]. The diagnostic criteria for fulminant type 1 diabetes had been made by the committee of the Japan Diabetes Society for fulminant and acute-onset type 1 diabetes mellitus [2]. In the diagnostic criteria, fulminant type 1 diabetes is confirmed when the following three findings are present: 1) the occurrence of diabetic ketosis or ketoacidosis soon (approximately seven days) after the onset of hyperglycemic symptoms, 2) plasma glucose level of ≥288 mg/dL and glycosylated hemoglobin (HbA1c) level of <8.7% at first visit, and 3) urinary C-peptide level of <10 μg/day or fasting serum C-peptide level of <0.3 ng/mL and <0.5 ng/mL after intravenous glucagon (or after meal) load at onset.

Typical fulminant type 1 diabetes shows irreversible insulin depletion after onset [3]. We experienced a case that followed a similar course to fulminant type 1 diabetes and then presented the recovery of insulin secretory ability.

## Case presentation

A 67-year-old woman experienced thirst, polyuria, loss of appetite, and weight loss. Because the symptoms persisted, she visited Otsu-Shiga Red Cross Hospital. Two months before the onset of the symptoms, she had not been pointed out to have impaired glucose tolerance in a medical checkup. Namely, HbA1c was 5.4%, and casual plasma glucose level was 86 mg/dL. She had a history of ischemic enteritis before the age of 63. She had no family history of diabetes. She did not drink alcohol but had smoked five cigarettes a day for 11 years before the age of 30. She drank about 300 mL of soft drinks daily. She lost weight from 48-49 kg to 45 kg after the onset of symptoms. Her non-fasting blood glucose level was 586 mg/dL, HbA1c was 7.2%, and the urinary ketone test was positive. She was then hospitalized for intensive insulin therapy. On day 2, blood tests showed increased total ketone bodies of 10,364 µmol/L, acetoacetic acid of 1,839 µmol/L, and 3-hydroxybutyric acid of 8,525 µmol/L. Arterial blood gas analysis showed metabolic acidosis, pH of 7.276, partial pressure of carbon dioxide (pCO_2_) of 22.9 mmHg, partial pressure of oxygen (pO_2_) of 86.9 mmHg, bicarbonate (HCO_3_) of 10.3 mmol/L, and base excess (BE) of 14.3 mmol/L. Her fasting C-peptide immunoreactivity (CPR) and postprandial CPR were 0.4 ng/mL (plasma glucose level of 278 mg/dL) and 0.5 ng/mL (plasma glucose level of 406 mg/dL), respectively. Her insulin secretion was not completely depleted but was markedly decreased. Anti-glutamic acid decarboxylase (anti-GAD) antibody and insulinoma-associated antigen-2 (IA-2) antibody were negative. Her serum amylase level was not elevated at 60 IU/L.

Abdominal plain computed tomography (CT) revealed a normal pancreas appearance. She was treated for fulminant type 1 diabetes. She maintained near-normal mealtime blood glucose levels (100-150 mg/dL) with insulin therapy at six units of insulin degludec before dinner, six units of insulin lispro before breakfast and lunch, and four units of insulin lispro before dinner. On day 25 after admission, she was discharged. Because of the tendency to hypoglycemia, the total daily dose of insulin was decreased from 22 units to eight units, that is, two units of insulin degludec before dinner and two units of insulin lispro before breakfast, lunch, and dinner. One month later, she was admitted to Kyoto University Hospital for the assessment of insulin secretory ability and further evaluation. On admission, her height and weight were 154.7 cm and 46.7 kg, respectively (body mass index of 19.5 kg/m^2^). There was no swelling or tenderness in the anterior neck. There was no hyperreflexia or hyporeflexia of the Achilles tendon reflexes and no decrease in vibration sense at the medial malleolus. There was no abnormal superficial sensation in the extremities. Fundus examination revealed no retinopathy. Laboratory findings on admission (Table 1) showed a fasting plasma glucose level of 106 mg/dL, HbA1c of 7.1%, and no urinary ketone bodies. Fasting and six-minute post-glucagon-stimulated CPR were 1.07 ng/mL (plasma glucose level of 106 mg/dL) and 2.04 ng/mL (plasma glucose level of 126 mg/dL), respectively. Daily urinary CPR was 62 µg. Unlike the finding at the previous hospitalization, her insulin secretory ability was not markedly decreased. Islet-specific autoantibodies, including anti-GAD antibody, IA-2 antibody, anti-insulin antibody, and anti-zinc transporter 8 (anti-ZnT8) antibody, were negative. The human leukocyte antigens (HLA) were DRB1*04:05:01 and DQB1*04:01:01. Proteinuria was negative. Daily urinary albumin was 4.9 mg. No diabetic microvascular complications were detected. Thyroid function was normal. Anti-thyroglobulin (anti-TG) antibody and thyroid peroxidase (TPO) antibody were negative.

**Table 1:** Laboratory findings on admission AMY, amylase; CPR, C-peptide immunoreactivity; Glu, glucose; HbA1c, glycosylated hemoglobin; Ab, antibody; GAD, glutamic acid decarboxylase; IA-2, insulinoma-associated antigen-2; ZnT8, zinc transporter 8; ALB, albumin; HLA, human leukocyte antigen

Parameter	Patient value	Reference range
AMY	68	44-132 U/L
Lipase	36	13-55 U/L
Fasting CPR	0.62	1.0-2.0 ng/mL
Fasting Glu	106	65-105 mg/dL
HbA1c	7.1	4.9%-6.0%
GAD Ab	<5.0	<5.0 U/mL
IA-2 Ab	<0.6	<0.6 U/mL
Insulin Ab	<0.4	<0.4 U/mL
ZnT8 Ab	<15.0	<15.0 U/mL
Urinary CPR	62	22.8-155.2 µg/day
Urinary ALB	4.9	<30 mg/day
Glucagon test		
CPR (zero minutes)	1.07	1.0-2.0 ng/mL
Glu (zero minutes)	106	65-105 mg/dL
CPR (six minutes)	2.04	>4.0 ng/mL
Glu (six minutes)	126	65-140 mg/dL
HLA DNA typing	DRB1*04:05:01 and DQB1*04:01:01	

She continued intensive insulin therapy (two units of insulin degludec before dinner and two units of insulin lispro before breakfast, lunch, and dinner) with a balanced diet providing 1,800 kcal of energy (target weight, 52.7 kg; activity factor, 34.2 kcal/kg). The mealtime blood glucose levels ranged from 80 to 130 mg/dL, and the postprandial blood glucose levels ranged from 110 to 200 mg/dL. We provided the patient with information regarding the coping method for hypoglycemia and sick days.

## Discussion

Fulminant type 1 diabetes is a subtype of type 1 diabetes characterized by the rapid onset and progression of islet cell destruction and the absence of islet-specific autoantibodies [1]. This case presented with symptoms of thirst, polyuria, and weight loss after a cold and developed into diabetic ketosis within a week. The casual blood glucose level and HbA1c at diagnosis were 288 mg/dL or above and below 8.7%, respectively. The islet-specific autoantibodies were negative. These clinical findings indicated a diagnosis of fulminant type 1 diabetes. While her fasting CPR and postprandial CPR levels were 0.4 ng/mL and 0.5 ng/mL, respectively, the slight increases did not reach the diagnostic criteria for fulminant type 1 diabetes of the committee of the Japan Diabetes Society for fulminant and acute-onset type 1 diabetes mellitus [2]. Also problematic for the diagnosis, her insulin secretory ability improved two months after treatment with insulin.

While typical fulminant type 1 diabetes shows irreversible insulin depletion after onset, in this case, insulin secretion was restored [3]. In fact, some cases of fulminant type 1 diabetes have been reported to exhibit residual insulin secretion (Table 2) [4-6]. As in this case, these cases showed colds as a prodrome and developed ketosis within four days after the appearance of the symptoms of hyperglycemia. These cases also showed hyperglycemia (blood glucose levels of 304-593 mg/dL), low HbA1c levels (5.5%-7.6%), and insulin secretory defect at onset. However, their fasting serum CPR was 0.61-1.94 ng/mL, and daily urinary CPR was 32-78.5 µg 1-10 months after diagnosis. Thus, although these cases were diagnosed as fulminant type 1 diabetes due to acute onset and insulin depletion at diagnosis, they exhibited improved insulin secretory capacity several months later. The mechanism of the restoration of impaired insulin secretion in these cases was postulated to be the early normalization of hyperglycemia at the onset of the disease that enabled the restoration of impaired pancreatic β-cells or the regeneration of severely damaged pancreatic β-cells [4-6].

**Table 2:** Overview of cases of diabetes following a course of fulminant type 1 diabetes with residual insulin secretion HbA1c, glycosylated hemoglobin; CPR, C-peptide immunoreactivity; HLA, human leukocyte antigen

Case	41-year-old woman	44-year-old woman	26-year-old man	67-year-old woman
Reference	[4]	[5]	[6]	Present case
Prodrome	Cold	Cold	Cold	Cold
Period after manifestation	4 days	0 days	0 days	3 days
Blood glucose level at onset	437 mg/dL	304 mg/dL	593 mg/dL	586 mg/dL
HbA1c level at onset	6.3%	5.5%	7.6%	7.2%
Urinary ketone body	(+)	(+)	(+)	(+)
Serum CPR at onset	(-)	Casual: <0.03 ng/mL	Fasting, 0.11 ng/mL; glucagon-loaded, 0.16 ng/mL	Fasting, 0.4 ng/mL; postprandial, 0.5 ng/mL
Urinary CPR at onset	9.5 µg/day	(-)	(-)	(-)
Serum CPR after onset	(-)	26 days: fasting, 1.94 ng/mL; postprandial, 4.27 ng/mL	10 months: fasting, 0.61 ng/mL; glucagon-loaded, 0.95 ng/mL. Two years: fasting, 0.59 ng/mL; glucagon-loaded, 1.22 ng/mL	2 months: fasting, 1.07 ng/mL; glucagon-loaded, 2.04 ng/mL
Urinary CPR after onset	1-12 months, 27.4-34.6 µg/day; five years, 14.7 µg/day	26 days: 78.5 µg/L		2 months: 62 µg/day
HLA	DRB1*04:05-DQB1*04:01 and DRB1*09:01-DQB1*03:03	DRB1*04:05-DQB1*04:01 and DRB1*08:03-DQB1*06:01	DRB1*04:05-DQB1*04:01 and DRB1*08:03-DQB1*06:01	DRB1*04:05-DQB1*04:01

In a clinical study comparing Japanese individuals having fulminant type 1 diabetes to healthy controls regarding the *HLA-DRB1* and *HLA-DRQ1* genes, which are associated with susceptibility to type 1 diabetes, those with fulminant type 1 diabetes had DRB1*04:05-DQB1*04:01 and DRB1*09:01-DQB1*03:03 with significantly higher frequency and DRB1*01:01-DQB1*05:01, DRB1*15:02-DQB1*06:01, and DRB1*08:03-DQB1*06:01 with significantly lower frequency than those without diabetes [7]. In addition, those with fulminant type 1 diabetes had DRB1*04:05-DQB1*04:01 with significantly higher frequency regardless of the homozygosity or heterozygosity [7]. Both the present case and the previous similar cases had DRB1*04:05-DQB1*04:01 (Table 2) [4-6]. The HLA DNA typing in this case did not differ from that in typical fulminant type 1 diabetes, as in the previously cited cases.

In the present case, it is necessary to differentiate a honeymoon period in acute-onset type 1 diabetes (idiopathic), soft drink ketosis, or ketosis-prone diabetes (KPD). A honeymoon period in acute-onset type 1 diabetes has been reported [8], but there are no previous reports of a honeymoon period in fulminant type 1 diabetes [9]. Acute-onset type 1 diabetes develops ketosis or diabetic ketoacidosis within three months after the onset of the symptoms of hyperglycemia. In addition, islet-specific autoantibodies are positive only at the rate of 18% in fulminant type 1 diabetes but are positive at the rate of 96% in acute-onset type 1 diabetes [10]. The present case developed diabetic ketoacidosis soon after the onset of the symptoms of hyperglycemia and had no islet-specific autoantibodies. Therefore, this case is unlikely to be considered acute-onset type 1 diabetes mellitus. Soft drink ketosis is a condition in which the overconsumption of soft drinks leads to a vicious cycle of polydipsia and soft drink intake and finally to diabetic ketoacidosis [11]. These cases have hyperglycemia and high HbA1c levels and require insulin therapy at onset due to decreased insulin secretory ability, but insulin therapy often becomes unnecessary later [11]. People with soft drink ketosis often have a habit of consuming over 2 L of soft drinks per day and exhibit obesity [11]. This case shows similarities to soft drink ketosis in that the decreased insulin secretory ability was restored after the initiation of insulin therapy. However, she drank only about 300 mL of soft drinks daily and did not exhibit obesity. In addition, this case had hyperglycemia but a low HbA1c level. Hence, this case had a low probability of soft drink ketosis.

KPD may represent an alternate underlying mechanism of the apparently idiopathic restoration of β-cell function in the present case. KPD was first reported in 1987 in a cohort of young African American men with obesity who developed diabetic ketoacidosis without an apparent cause [12]. Although they exhibited markedly impaired insulin secretion and insulin action at onset, intensive diabetic management improved pancreatic β-cell function and insulin sensitivity, often allowing the discontinuation of insulin therapy within several months [12]. KPD has also been reported in Asian individuals [13], in whom it is found most frequently in obese young men with a family history of diabetes and nearly new-onset diabetes [12,13]. Although the present case has similarities to KPD in terms of acute onset and subsequent improvement of insulin secretory ability, it is distinct in several notable ways, including gender, weight, and family history. From these considerations, the present case is most likely fulminant type 1 diabetes.

## Conclusions

Some cases of fulminant type 1 diabetes improve insulin secretory capacity, like this case and the previous similar cases. It is suggested that fulminant type 1 diabetes presents with a variety of clinical conditions including insulin secretory capacity. A case series of fulminant type 1 diabetes with residual insulin secretion will be needed for further verification.

## References

[1] Imagawa A, Hanafusa T, Miyagawa J, Matsuzawa Y (2000). A novel subtype of type 1 diabetes mellitus characterized by a rapid onset and an absence of diabetes-related antibodies. Osaka IDDM Study Group. N Engl J Med.

[2] Imagawa A, Hanafusa T, Awata T (2012). Report of the committee of the Japan Diabetes Society on the research of fulminant and acute-onset type 1 diabetes mellitus: new diagnostic criteria of fulminant type 1 diabetes mellitus (2012). J Diabetes Investig.

[3] Shibasaki S, Imagawa A, Terasaki J, Hanafusa T (2010). Endogenous insulin secretion even at a very low level contributes to the stability of blood glucose control in fulminant type 1 diabetes. J Diabetes Investig.

[4] Tanaka T, Morioka K, Tsuji M, Misaki M (2002). [Improved insulin secretion in fulminant type 1 diabetes mellitus] (Article in Japanese). Diabetes.

[5] Yamashita K, Sato Y, Seki K, Asano J, Funase Y, Yamauchi K, Aizawa T (2013). Fulminant type 1 diabetes with robust recovery of insulin secretion: a case report. Diabetes Res Clin Pract.

[6] Kaneko K, Satake C, Yamamoto J (2017). A case of idiopathic type 1 diabetes with subsequent recovery of endogenous insulin secretion despite initial diagnosis of fulminant type 1 diabetes. Endocr J.

[7] Tsutsumi C, Imagawa A, Ikegami H, Makino H, Kobayashi T, Hanafusa T (2012). Class II HLA genotype in fulminant type 1 diabetes: a nationwide survey with reference to glutamic acid decarboxylase antibodies. J Diabetes Investig.

[8] Zhong T, Tang R, Gong S, Li J, Li X, Zhou Z (2020). The remission phase in type 1 diabetes: changing epidemiology, definitions, and emerging immuno-metabolic mechanisms. Diabetes Metab Res Rev.

[9] Hanabusa T, Imagawa A, Iwahashi H (2005). [Report of Japan Diabetes Society on fulminant type 1 diabetes mellitus research: epidemiological and clinical analysis and proposal of diagnostic criteria] (Article in Japanese). Diabetes.

[10] Kawasaki E (2023). Anti-islet autoantibodies in type 1 diabetes. Int J Mol Sci.

[11] Yamada K, Nonaka K (1996). Diabetic ketoacidosis in young obese Japanese men. Diabetes Care.

[12] Umpierrez GE, Smiley D, Kitabchi AE (2006). Narrative review: ketosis-prone type 2 diabetes mellitus. Ann Intern Med.

[13] Balasubramanyam A (2019). Syndromes of ketosis-prone diabetes. Trans Am Clin Climatol Assoc.

